# Adherence to Immunosuppressive Therapies after Kidney Transplantation from a Biopsychosocial Perspective: A Cross-Sectional Study

**DOI:** 10.3390/jcm11051381

**Published:** 2022-03-02

**Authors:** Justyna Zachciał, Izabella Uchmanowicz, Magdalena Krajewska, Mirosław Banasik

**Affiliations:** 1 Department of Nephrology and Transplantation Medicine, Wroclaw Medical University, 50-556 Wroclaw, Poland; zajustyna@o2.pl (J.Z.); magdalena.krajewska@umw.edu.pl (M.K.); miroslaw.banasik@umw.edu.pl (M.B.); 2 Department of Clinical Nursing, Wroclaw Medical University, 51-618 Wroclaw, Poland

**Keywords:** kidney transplantation, medical adherence, immunosuppressive medication, acceptance of illness, frailty, quality of life, demographics

## Abstract

Kidney transplantation (KT) is the best method for kidney replacement therapy (KRT) because of patient survival rates and quality of life (QoL). Nowadays, the main cause of graft loss is antibody-mediated rejection. The treatment of humoral injury is difficult with uncertain results and still not firmly established. Therefore, appropriate adherence is crucial to prolong graft and patient survival. This study aims to evaluate the association of transplant patients’ acceptance of illness, symptoms of anxiety and depression, frailty, and QoL with medication adherence in KT recipients. A total of 210 patients after KT completed the surveys. The instruments were distributed during patients’ admission at the clinic by a qualified nurse, who assisted the patients’ in completing the questionnaires. A cross-sectional study of KT recipients 9.45 ± 7.26 years after KT was performed. Patient adherence with medications was assessed using the Adherence to Refills and Medications Scale (ARMS). Explanatory variables were examined with validated instruments, such as the World Health Organization Quality of Life (WHOQoL-BREF) questionnaire, The Mini-Mental State Examination (MMSE), the Acceptance of Illness Scale (AIS), the Hospital Anxiety and Depression Scale (HADS), and the Tilburg Frailty Indicator (TFI) scale, respectively. Simple linear and multiple regression analyses demonstrated the positive correlation between acceptance of illness and adherence to immunosuppressive medications in a patient sample of KT recipients. The other important factor facilitating adherence to medications was linked with physical and environmental dimensions. On the other hand, frail kidney transplant patients were more likely to be non-adherent. In conclusion, identifying contributors to better medication adherence in immunosuppressive therapy is crucial in preventing transplant rejection or graft loss. In the kidney transplant population, the acceptance of illness, selected dimensions of QoL, and demographic variables associated with rural living and vocational education favored adherence behaviors.

## 1. Introduction

Non-adherence is a major risk factor for rejection and allograft loss among transplant recipients [1,2,3]. Medical reports on non-adherence to immunosuppressive therapies after a transplant state that non-adherence is more prevalent than previously assumed. A variety of reasons for non-adherence occurrence may cause difficulty in its relevant recognition and assessment. Moreover, even though non-adherence in a population of transplant recipients is costly on several levels (for instance, social, medical, and economic), it is resistant to change from a behavioral perspective [4].

More specifically, recent data on the frequency and impact of non-adherence to immunosuppressants after kidney transplantation (KT) indicates that 22% of recipients was non-adherent, and 36% of graft loss was associated with non-adherence [5]. Non-adherent patients were seven times more at risk of graft failure than adherent patients [5]. Even minor deviations from the immunosuppressive medication dosing schedule may increase the risk of late acute rejection [6]. Nevins and Thomas [7] monitored medication adherence in 180 newly transplanted patients using electronic medication event monitoring system (MEMS) technology. Remarkably, after 4 years of gaining the data from monitoring the transplanted patients, with a subsequent 4.7 year follow-up, the authors concluded that minor medication non-adherence might have predictive values for worse clinical outcomes in transplant recipients [7].

The importance of strict adherence to immunosuppressive medication in the post-transplant phase has been investigated in many studies [5]. Current research must focus on identifying and measuring potential factors affecting adherence, both in positive and negative ways. Establishing the risk and protective factors of non-adherence in the population of transplant recipients could be worthwhile for successful intervention to improve adherence and, in consequence, significantly improve transplant outcomes [8].

The most important sociodemographic and medical factors affecting adherence negatively include variables, such as patient age, the complexity of the immunosuppressive regimen, side effects of medication, poor understanding of recommendations, and longer period from transplantation [9,10] as well as psychosocial variables, such as depression [11], anxiety [12], social functioning [13], and transplant-related stress [14].

The health-related quality of life (HRQoL) is a concept broadly studied in patients with chronic diseases. Researchers also pointed at the quality of life (QoL) factor that is significantly better in transplant patients than in chronic hemodialysis patients, and QoL reaches similar levels to the general population. In turn, HRQoL seems to be stable among transplant patients between one and two years after transplantation [15]. The HRQoL construct includes several dimensions linked with the patients’ perspectives and captures well their health-related outcomes. However, to date, measures of the HRQL parameter were performed only in a limited number of clinical studies investigating subjective health and the effectiveness of immunosuppressive therapies [16,17].

Although the advantages of KT for the QoL in comparison to treatment for end-stage renal disease are well established [18,19], there are still many difficulties faced by KT recipients after transplant [20,21,22]. Patients’ lives after KT also presents negative aspects, such as a strict regimen of immunosuppressive drugs and their related side effects, frequent medical visits and hospitalizations, infections, and symptoms of anxiety and depression concerning rejection episodes and the potential loss of the graft [23,24,25,26]. It is worth noting that after KT and discharging the patients with a functioning transplant, their life with chronic illness is continued [27]. The findings reveal that the transplant recipients still have several medical problems, and their life requires several adjustments [27,28,29]. Therefore, it seems that interventions addressing HRQoL in the post-transplant populations should become a priority also because the low HRQoL may be a predictive factor for non-adherence.

The hypothesis that HRQoL may be the critical predictor of adherence is supported by similar research outcomes in the other populations of chronically ill patients. For example, poor HRQoL significantly predicted treatment discontinuation in chronic hepatitis C patients [30]. As mentioned above, adherence may be affected by several biological and psychosocial factors. Therefore, in addition to HRQoL, sociodemographics, and medical conditions, other significant predictors of adherence should be included, such as acceptance of the illness, frailty, and the patients’ cognitive impairment.

To date, associations between the acceptance of illness and adherence level have not been thoroughly investigated among patients after KT. In general, a higher acceptance of the illness increases patients’ adaptation to the disease and reduces their psychological distress, including negative emotions and reactions [31]. A recent study by Fedorowicz et al. [32] showed that an increased acceptance of illness in kidney graft patients predicted a lesser extent of comorbidities and lower interest in receiving health services. Nonetheless, the above study did not correlate the Acceptance of Illness Scale (AIS) scores with a standardized measure of adherence. A recent report on patients undergoing immunosuppressive treatment suggests the association between acceptance of illness that considers a new definition of adherence [33]. This research proposes that the definition of “non-adherence by non-acceptance” implicates that “the patient does not accept the disease and refuses to continue treatment” [33]. This strictly implies that acceptance of the illness positively affects adherence behavior in kidney transplant recipients.

Measurements of adherence to prescribed medication are challenging because self-reports are not without methodological constraints [34]. This is partly due to the difficulty of operationalizing medical adherence and the evolution of its relevant terminology over time [35]. Considering the recent conceptualization of medication adherence, the Adherence to Refills and Medications Scale (ARMS) [36] seems to appropriately fit into the taxonomy of “adherence to medications” and “management of adherence” components. Kripalani et al. [36] developed ARMS for patients with a chronic condition (i.e., coronary heart disease). Initial material of the original ARMS was created by a team with multidisciplinary expertise and based on a literature review for ranges referring to self-reported medication (i.e., the Morisky Medication Adherence Scale, MMAS, and the Hill–Bone Medication Adherence Scale, HBMAS). The resulting 12 items in the final survey included 2 subscales: an adherence to take medications (8 items) and an adherence to refill prescriptions (4 items). The ARMS was utilized amongst patients with chronic diseases [37]. The ARMS is a legitimate and reliable medication adherence instrument used in a population with a chronic condition, with good performance characteristics amongst low-literacy people [36].

Following the view of “naturalistic decision-making,” self-care behaviors, including adherence, implicate the processes a person engages to maintain health and manage acute and chronic illness [38]. This approach assumes that adherence depends on the patients’ cognitive abilities, including their memory, general intellectual ability, and organization skills [39]. It is well established that patients experiencing cognitive and emotional problems are more likely to have difficulties following prescribed treatment regimens [40,41,42,43]. For example, a study in the kidney transplant recipients indicated that poorer everyday problem-solving and more depressive symptoms were predictive of low medication adherence [44]. Frailty is another factor significantly associated with cognitive impairment [45] that may be essential for adherence in the kidney transplant population. This syndrome is defined by five criteria: slowness, weakness, low physical activity, exhaustion, and shrinkage [46]. Recently, frailty has also been associated with self-care behaviors in several chronic diseases [47].

The main objective of this study is to explore the risk and protective factors associated with poor adherence and the relationships between adherence and clinical outcomes in a Polish population of transplant recipients. Firstly, we studied essential sociodemographic factors about the patients and their clinical and psychological characteristics, including acceptance of illness, symptoms of anxiety and depression, and the information on how patients perceive their QoL parameters after a transplant. Secondly, we verified the hypothesized links between the relevant factors and adherence.

## 2. Materials and Methods

### 2.1. Participants and Design

Patients who visited the outpatient clinic of the University Clinical Hospital in Wroclaw (Poland) between 2018–2020 were recruited to the study. The study included patients who were 18 years old and older on the day of the study and diagnosed with end-stage chronic kidney disease (CKD) treated with a kidney transplant. The study was a cross-sectional observational study; thus, the Strengthening the Reporting of Observational Studies in Epidemiology (STROBE) guidelines were followed.

### 2.2. Ethical Considerations

The study protocol was approved by the Bioethics Committee of the Wroclaw Medical University (approval no. KB-789/2018). Participation in this study was voluntary and anonymous. Before participating in the survey, respondents were presented with the essential concerning the study. All patients gave their informed consent to participate in the study and were then asked to complete the questionnaires during hospitalization.

### 2.3. Instruments

The Polish version of the Tilburg Frailty Indicator (TFI) scale was used in the study [48]. The scale comprises two parts. The first part measures determinants of frailty, including the participant’s sociodemographic characteristics (sex, age, marital status, country of origin, educational level, and monthly income), lifestyle, multimorbidity, life events, and living environment. The second part measures three domains. The physical domain (0–8 points) includes 8 items related to physical health, unexplained weight loss, difficulty in walking, balance, hearing problems, vision problems, strength in hands, and physical tiredness. The psychological domain (0–4 points; 4 items) evaluates cognition, depressive symptoms, anxiety, and coping. Finally, the social subscale (0–3 points) assesses the social aspects of the patients’ lives in terms of living alone, lack of social relations referring to loneliness, and no social support. Frailty is diagnosed when the overall TFI score is ≥5. The instrument was adapted and translated for the Polish cultural setting by Uchmanowicz et al. [48] and demonstrated the satisfactory Cronbach’s alpha internal consistency of 0.74.

The Mini-Mental State Examination (MMSE) developed by Folstein et al. [49] is a common screening test for dementia; it is rapid to complete and easy to interpret. The administration and scoring procedures of the measurement are easily learned; the administration of the test takes about five to ten minutes. The MMSE enables the assessment of cognitive functions in perception, memory, attention, linguistic functions, visual-spatial abilities, and the ability to count, recall things, repeat, and carry out orders. The maximum MMSE score is 30 points—the lower scores indicate severe cognitive dysfunction. Patients who scored less than 23 points are diagnosed as cognitively impaired.

The AIS measurement originally developed by Felton et al. [50] and adapted to Polish conditions by Jurczyński [31] was also applied. The AIS uses eight items that measure impairments imposed by the illness, a lack of independence due to the illness, the feeling of dependency, and reduced self-esteem. Participants were asked to indicate their feelings on a 5-point Likert scale from 1 (“strongly agree”) to 5 (“strongly disagree”). The total AIS score ranged from 8 to 40; the higher score, the better the acceptance of illness.

The WHOQOL-BREF is a popular short version of WHOQoL-100 that comprises 26 items assessing the QoL in 4 domains: physical, psychological, social, and environmental. In addition, the WHOQoL-BREF includes two supplementary items evaluating the overall perception of QoL and health. Higher scores denote a higher QoL. The Polish version of the questionnaire demonstrates good internal consistency, reflecting the reliability of the Cronbach’s alpha coefficients higher than 0.70 for 3 domains, and 0.69 for the social domain [51].

The Hospital Anxiety and Depression Scale (HADS) developed by Zigmond and Snaith [52] evaluates the levels of anxiety and depression based on 16 statements rated by the patient using a 4-item scale (0–3). The questionnaire has two subscales of anxiety and depression. Each subscale includes 7 items that are rated at 0–3 points. Respondents with subscale scores of 0–7 are considered normal, while scores of 8–10 denote mild abnormality, 11–14 moderate abnormality, and 15–21 severe abnormality.

The ARMS for assessment of adherence was first used by Kripalani et al. [36] in patients with coronary artery disease and other chronic conditions, including hypertension, dyslipidemia, and diabetes mellitus. Overall, the inventory includes 12 items gathered in 2 subscales: adherence to taking medications (8 items) and adherence to refilling prescriptions (4 items). The responses were scored on a 4-point Likert scale from 1 (“none”) to 4 (“all of the time”). The item scores are summed to an overall score (from 12 to 48 points), with the lower scores indicating better adherence [53].

### 2.4. Statistical Analysis

Quantitative analysis was performed by calculating the mean (M), standard deviation (SD), median, quartiles, minimum, and maximum. The statistical characteristics of the qualitative variables are shown as frequencies (n and %). Then, simple linear and multiple analyses were performed using the linear regression method. The normality distribution of residuals for regression was examined with the Shapiro–Wilk test; no significant departures from normality were indicated with this test. The Breusch–Pagan test was used to test for heteroskedasticity in the linear regression models.

VIFs underwent standard statistics to evaluate the artificial inflation of the variance for a regression coefficient. A rule of thumb is that a VIF value greater than 10 indicates multicollinearity. The higher VIF indicates a more problematic f collinearity between predictors. The 95% confidence intervals (CI) of the regression coefficients were estimated for the proposed linear regression models. The quality of the resulting models was assessed by calculating the R^2^ determination coefficients. For all analyses, the statistical significance was set at *p* < 0.05. Statistical analyses were carried out with the R Statistical Package, version 4.0.3 [54].

## 3. Results

### 3.1. Examination of Heteroskedasticity and Multicollinearity in the Linear Regression Models

The Breusch–Pagan test indicated that the homoscedasticity was present in each linear regression model: for the general ARMS scale, B–P (31) = 23.86, *p* = 0.816; ARMS taking medications scale, B–P (31) = 38.20, *p* = 0.175; and ARMS drug supplementation and refill scale, B–P(31) = 27.41, *p* = 0.651. We then detected the degree of multicollinearity between the predictors in the regression models by analyzing the VIF factors. For instance, Hair et al. [55] suggested that VIFs greater than 4 indicate possible multicollinearity. In this study, the maximum VIF was 3.526 (see the VIFs in Table 1). These results indicate no multicollinearity among the explanatory variables.

### 3.2. Sociodemographic and Clinical Characteristics

The study involved 210 patients, including 97 women (46.19%) and 113 men (53.81%). The mean age of the patients was 59.88 years (SD = 13.23). The median value indicated that half of the patient sample was less than 64 years of age. The mean time to transplant was 9.45 years (SD = 7.26), with a median of 8 years. Most of the respondents lived in the city (70.48%) and were married (77.62%). Among the respondents, 7.14% of them had only primary education. Vocational education was held by 29.05% of the patients, 38.57% of the respondents had secondary education, while higher education was declared by 25.24% of the people. Most respondents were retirees (48.57%) and pensioners (31.43%); note that pensioners are individuals who receive sickness benefits without reaching retirement age. Only 18.10% of patients were employed; 1.9% of the respondents declared unemployed. The most common comorbidity was hypertension, which was reported by 81.43% of the patients. The second most prevalent comorbidity was diabetes mellitus, which affected 23.33% of the sample. The rarest of the coexisting diseases was hypercholesterolemia, which was present in 10.48% of the patients. The concentration of tacrolimus < 5 mg or cyclosporine < 100 mg was observed in 62.38% of the patients, of whom 23.81% declared that it rarely reached these levels, whereas 15.71% of patients reported that it happened to occur sometimes. The high frequency of incidences (often and very often) of both the tacrolimus concentration < 5 mg or cyclosporine < 100 mg was observed in 48 cases (see Table 2).

### 3.3. Psychosocial Factors

Concerning the psychosocial factors, KT recipients were evaluated with the quantitative measures of symptoms of depression and anxiety (HADS), acceptance of illness (AIS), and mental ability (MMSE). In general, 3.33% of the patients in this study had clinically relevant depressive symptoms (HADS depression score, >10) and 9.52% clinically relevant anxiety symptoms (HADS anxiety score, >10). In addition, the AIS score range is in the psychosocial factor of 8–40 points, and the higher the score, the greater the acceptance of illness. The mean AIS score was 30.49 points, which is 3.81 points per question, which meant that the respondents tended to accept their illness.

The MMSE scores were used to diagnose mental ability in the patient sample. It turned out that 126 out of 210 survey participants (60.00%) had a normal mental ability score, 59 respondents (28.10%) exhibited cognitive impairments without dementia, while 24 respondents (11.43%) had mild dementia, and 1 respondent (0.48%) appeared to have moderate dementia.

The ARMS questionnaire was used to access adherence behaviors. The mean score for overall ARMS was 14.71 (SD = 1.87). The third quartile (Q3) value indicated that 75% of the patient sample scored less than 15 points. That is, 75% of the patient sample exhibited high adherence behaviors. The mean values for subscales (i.e., taking medications and drug supplementation and refills) were 8.65 (SD = 1.33, Me = 8) and 6.06 (SD = 1.31, Me = 7), respectively.

### 3.4. ARMS Overall Adherence: Simple Linear Regression and Multiple Regression Analyses

The results of a series of simple linear regression analyses are presented in Table 3. An important predictor of the total ARMS result was the residence. Living in the countryside was associated with the lower ARMS score by an average of 0.709 points compared to the patients living in the city, B = −0.709, 95% CI: [−1.256; −0.162]; *p* = 0.012. Among the important predictors of adherence improvements, there was also the level of disease acceptance (AIS). We found that the higher the AIS score, the lower the overall ARMS score, B = −0.035, 95% CI: [−0.066; −0.005]; *p* = 0.025. Additionally, the results of simple linear regression analysis indicate that the higher level of QoL in the dimension of health perception is associated with the improvements of adherence (B = −0.251, 95% CI: [−0.495; −0.006]; *p* = 0.046). An increase in the physical domain of QoL by one point was associated with the lower ARMS result by an average of 0.108 points, B = −0.108, 95% CI: (−0.201, −0.014); *p* = 0.025. Similarly, a 1-point increase in the QoL in the psychological domain decreased the ARMS score by an average of 0.113 points, B = −0.113, 95% CI: [−0.22; −0.006]; *p* = 0.039. The significant predictor of adherence turned out to be the environmental dimension of QoL. The increase of 1 point on the WHOQoL-BREF scale in this domain was associated with a decrease in the ARMS score by a 0.167 point, B = −0.167, 95% CI: [−0.285; −0.048]; *p* = 0.006. On the other hand, the regression analysis indicated that the severity of frailty syndrome significantly predicted worsening in adherence, B = 0.094; 95% CI: [0.004, 0.185]; *p* = 0.041, because 1 point higher on the TFI score was associated with the increase in the ARMS score by 0.094 points on average.

In the next step, the multiple regression model was performed to evaluate how all the considered predictors affected the overall adherence at the same time. It turned out that only the place of residence significantly predicted improvement of overall adherence to the doctor’s recommendations. We found that rural living was associated with decreasing the ARMS score by 0.733 points on average, B = −0.733, 95% CI: [−1.388, −0.079]; *p* = 0.029.

### 3.5. ARMS Taking Medications Subscale: Single and Multiple Regression Analysis

The simple linear regression analysis (see Table 3) showed that hypercholesterolemia was an important predictor of non-adherent drug intake. The co-morbidity of hypercholesterolemia was associated with the increase in ARMS taking medications by a 0.81 point on average, B = 0.81, 95%CI: [0.219; 1.38]; *p* = 0.007. On the other hand, another significant predictor for improving taking medicines as initially hypothesized was the higher level of AIS. A 1-point increase in the AIS score was associated with a decrease in the ARMS taking medications by 0.046 points, B = −0.024, 95% CI: [−0.046, −0.002]; *p* = 0.037. Only a physical dimension of QoL significantly predicted better adherent outcomes of drug intake. The results of the simple linear regression analysis show that 1 point higher on the WHOQoL subscale in the physical domain is associated with the lower ARMS taking medications by 0.09 points, B = −0.09, 95%CI: [−0.157; −0.024]; *p* = 0.008. The severity of the psychological dimension of frailty syndrome was shown to be predictive of a worsening patient drug intake. A 1-point increase in the psychological dimension of FS syndrome was associated with the mean increase in ARMS taking medications score by 0.193 points, B = 0.193, 95%CI: [0.01; 0.374]; *p* = 0.039. The multiple linear regression model showed that the MMSE score was also an important predictor of a non-adherent outcome for patients’ activity of taking medications, because the increase in the MMSE by 1 point predicted the increase in the ARMS taking medications score by 0.04 points on average, B = 0.084, 95% CI: [0.008; 0.16]; *p* = 0.032.

### 3.6. ARMS Drug Supplementation and Refill: Single and Multiple Regression Analyses

The simple linear regression analysis (see Table 4) indicated that an important predictor of adherence was the environmental domain of QoL. It turned out that the increase in the WHOQoL-BREF subscale in the environmental dimension was associated with a lower score on the ARMS subscale by an average of −0.094 points, B = −0.094, 95% CI: [−0.177; −0.011], *p* = 0.028. On the other hand, an increase in the social component of frailty scale by 1 point was associated with the increase in the ARMS score (drug and prescription supplementation) by an average of 0.276 points, B = 0.276, 95% CI: [0.019; 0.534]; *p* = 0.037.

The multiple regression analysis of the ARMS (taking medications and refilling prescriptions—see Table 5) showed that the independent predictor was education. Compared to patients with primary education, patients with a vocational education had, on average, a lower ARMS drug supplementation and refill score by 0.859 points, B = −0.859, 95% CI: [−1.651; −0.067]; *p* = 0.035. In addition, comorbidity was also the independent predictor for non-adherent behavior as patients with hypercholesterolemia scored significantly lower by 0.814 points on the ARMS subscales (taking medications and refilling prescriptions), B = −0.814, 95% CI: [−1.458, −0.171]; *p* = 0.014. The independent predictor of the improvements of taking medications and refilling prescriptions was the level of the environmental QoL as a 1-point increase in the WHOQoL subscale was associated with the mean decrease of 0.129 points on the ARMS score, B = −0.129, 95% CI: [−0.256; −0.002]; *p* = 0.049.

## 4. Discussion

Identifying the contributors to low medication adherence in immunosuppressive therapy is crucial in preventing transplant complications or graft loss. In our study, almost all participants showed satisfactory adherence to their immunosuppressive treatment in both subscales in the ARMS, with a medication refill subscale slightly worse than adhering to the pill-taking regimen. This seems to be an interesting finding since currently reported adherence rates after an organ transplant in the literature are much lower. How do we substantiate these findings concerning the other factors we studied? Concerning the sociodemographic factors, non-adherence is often linked to young age. Pinsky et al. [2] found that adolescent recipients aged 19 to 24 years were more likely to have persistent noncompliance than patients aged 25 to 44 years. The mean age of patients involved in our study was 60 years. Thus, our sample was relatively old. Our results seem consistent with the finding that older patients after kidney replacement therapy (KRT) show better compliance to prescribed oral medication than their younger counterparts [56]. However, the present findings are based on a cross-sectional design; therefore, drawing more general conclusions would require a longitudinal and multi-level perspective.

The present study investigated necessary adaptation processes to chronic illness, such as adjustment and acceptance of illness that represent major psychosocial factors influencing KRT [57]. We quantified this psychosocial factor with a survey measurement based on Felton and Revenson’s construct [50]. The survey directly measures how the patient can accept his or her illness with no experiences of negative emotions and behavioral responses [50]. It positively influences the complex therapeutic regimens of immunosuppressive therapy that significantly burdens KT patients [57]. Little research investigates the effects of acceptance of illness on adherence in populations of patients with kidney diseases, and the research on coping styles with chronic illness in this patient population seems to be underestimated [12].

Nonetheless, some reports suggest that a population of patients with functioning renal transplants tend to have higher AIS scores than patients on hemodialysis or peritoneal dialysis [57], but acceptance of the illness in patients after KT has been not analyzed as a predictive factor of adherence. Although some researchers suggest that no acceptance of the illness can cause refusal of the treatment [33], our study showed that patients with functioning renal transplants with higher AIS scores presented better adherence behaviors. However, one should remember that this result is significant only if the regression model does not consider other predictors. Other variables might have served as mediators in the relation between AIS and adherence. In addition, it turned out that patients with renal transplants who were employed and had full-time education also were more effectively able to adapt to their chronic conditions [57].

It is also worth mentioning that the association between adherence and QoL has not extensively been studied in KT patients. To date, few reports show that QoL is recognized as a key factor for successful KT [58,59]. In general, clinical reports established that either patients’ perceptions of health, psychological domain, social functioning, or the environmental dimension was predictive of the overall improvement to adherence in immunosuppressive therapies [12,60]. The findings from the present study are in line with recent reports [12,59] showing that the higher levels of QoL in the psychological, subjective perception of patients’ health in the physical and environmental domains can be positively correlated with adherence improvements. However, when we put all predictors in the regression model, the QoL variable was no significant predictor of overall adherence. Therefore, the relationship between QoL may be mediated by the other included variables.

Interestingly, the present study did not find the association between depression and adherence behaviors. Symptoms of depression are considered significant risk factors that are highly associated with non-adherence in CKD. For instance, Cukor et al. [61] investigated a correlation between depression and adherence to immunosuppressant medications in kidney transplant recipients. This study found that higher depression levels correlated with missing more medication doses. Jindal et al. [62], using the United States Renal Data Service data in 2009, conducted a retrospective cohort study of 32,757 Medicare primary KT recipients and showed a strong association between depression and non-adherence, regardless of whether the symptoms of depression were diagnosed in the pre- or post-transplant stage [63]. Intriguingly, a recent report by Scheel et al. [59] indicated no association between symptoms of depression and anxiety and adherence. This effect was explained by the relatively small proportion of depressive patients (16.7%) with an elevated HADS depression score (HADS > 10), which is, in fact, much higher than the proportion found in the present study (3.33% with HADS depression score, >10).

The ARMS scale has been shown to be an objective measure of adherence and might resolve the constraints of existing self-report medication adherence measures [64]. Our findings show that ARMS identifies the barriers to adherence in KT patients well, which might be useful in research study and clinical practice in kidney transplantation. The most extensively utilized self-report measurements of immunosuppressive medication adherence in kidney transplant recipients are the MMAS [65] and also the Medication Adherence Report Scale (MARS) [66]. Although these self-reports measures work for simple and quick evaluations of medication adherence, they appear to not supply enough information regarding adherence to targeted interventions to chronic patients for non-adherence or low adherence [37]. This is mainly as a result of minimal information acquired (i.e., single measurement or prescription refills) and limited response ranges (i.e., dichotomous scale) [37]. It should be noted that ARMS was developed and administered to patients with coronary artery disease (CAD) in an inner-city primary care clinic [36]. Hence, this conceptualization of medication adherence as a standard for reporting medication adherence research may be debatable given the ABC taxonomy [35].

Several recent post-transplant follow-up reports show that kidney recipients with lower perceived social support, lower cognitive abilities, and younger age are considered to be at risk for immunosuppressive medication non-adherence [59]. Our cross-sectional study provides similar evidence that kidney recipients with lower cognitive abilities, more prone to frailty syndrome, and symptoms of hypercholesterolemia are more likely to present non-adherent behaviors. Indeed, frailty is a risk factor for early hospital readmission [67] and delayed graft function in KT recipients [68]. Thus, frailty syndrome may cause complications and worse outcomes via non-adherence in KT recipients. Interestingly, comorbid patients with symptoms of hypercholesterolemia exhibit opposite behavioral patterns in both ARMS subscales. In particular, the analysis indicated that hypercholesterolemia in these patients seems to contribute to their failure to take medication. On the other hand, it seems to encourage better refill adherence (when other variables are included in the regression model). Thus, the understanding contribution of comorbidity of hypercholesterolemia in kidney recipients should be of great importance, as this sort of medication non-adherence can lead to excess morbidity and mortality in this group of patients.

### 4.1. Study Limitations

The present study has limitations. The first is limited clinical significance because the modeling results obtain values ranging from 0.05 to 1.5 points on the ARMS scale ranging from 12 to 48 points. Therefore, the clinical importance may be debatable. Further research with larger samples and multicenter studies are needed to confirm the clinical significance of the identified factors in this study. A second limitation arises from operationalizations of adherence that were used in the construction of the ARMS instrument. It cannot be ruled out that some of the ARMS items are non-relevant for the KRT patient (for example, see the analysis of the validity and reliability for diabetes patients proposed by Mayberry et al. [64]). The next step should then be analyses of construct and predictive validity of the ARMS and consensus analysis of terminology and taxonomy of patient compliance. Additionally, it should be emphasized that other important outcomes, such as the graft kidneys, either renal function or kidney rejection, should be considered in further studies. A crucial aspect in terms of the impact of sociodemographic variables on medication adherence, which was missing in our study, is the analysis of how the material status and income of KT patients may affect the coverage of treatment costs, in particular, the purchase of prescribed immunosuppressive medications. However, it should be pointed out that this is not a problem in the country where the study was conducted (Poland), as the cost of medications is refunded by the national health care system. Immunosuppressive medications are refunded with a patient payment of a nominal cost of one dollar per medication pack.

### 4.2. Practical Implications

Characterizing patient adherence profiles should be performed to identify the barriers to adherence proposed by adapted therapeutic, educational programs. Regularly marking adherence, afterwards, may help to prevent the multiplication of non-adherence episodes in patients with a poor adherence profile early on and the occurrence of non-adherent episodes in patients initially considered to be adherent by organizing individualized and targeted interventions.

The improvement of patient care should include drug regimen optimization, close adverse events management, and consideration of social, psychological, and biological factors. Psychological aspects after transplantation should never be neglected, and the influence of the mental component of QoL and depressive symptoms on adherence should not be underestimated. Future research should verify whether multidisciplinary teams, including allied professionals, and psychological and behavioral interventions can improve adherence.

## 5. Conclusions

The acceptance of illness is associated with an adherence to immunosuppressive medications in a patient sample of KT recipients. Another important factor facilitating adherence with medications is QoL, especially the physical and environmental dimensions. Frail kidney transplant patients were more likely to be non-adherent; therefore, identifying contributors to improve medication adherence in immunosuppressive therapy is crucial in preventing transplant rejection or graft loss. In the kidney transplant population, the acceptance of illness, selected dimensions of QoL, and demographics associated with rural living and vocational education favored adherence behaviors.

## Figures and Tables

**Table 1:** Variance inflation factors (VIFs) measure the amount of multicollinearity in the regression models.

Independent Variables in the Regression Models		VIF
Gender		1.254
Age		2.777
Place of residence		1.411
Civil status		1.342
Education		1.890
Professional activity		3.479
Time since transplant		1.442
Comorbidities	Diabetes	1.185
	Hypercholesterolemia	1.272
	Hypertension	1.328
	Rheumatic diseases	1.358
	Other	1.195
Blood concentration of tacrolimus < 5 mg or ciclosporin < 100 mg		2.007
AIS (total score)		1.980
HADS	Anxiety	2.320
	Depression	3.120
WHOQoL-BREF	Perception of quality of life	1.550
	Perception of health	1.652
	Physical dimension	3.186
	Psychological dimension	3.526
	Social dimension	2.349
	Environmental dimension	2.350
TFI (total score)		1.798
MMSE		1.447

Abbreviations: VIF, variance inflation factor; AIS, Acceptance of Illness Scale; HADS, Hospital Anxiety and Depression Scale; WHOQoL-BREF, World Health Organization Quality of Life—Short Form; TFI, Tilburg Frailty Indicator; and MMSE, Mini-Mental State Examination.

**Table 2:** Characteristics of respondents.

Parameter			Total (*n* = 210)
Age (years)		Mean ± SD	59.88 ± 13.23
		Median	64
		Quartiles	52–69
Time since transplant (years)		Mean ± SD	9.45 ± 7.26
		Median	8
		Quartiles	3–15.75
Gender		Women	97 (46.19%)
		Men	113 (53.81%)
Place of residence		City	148 (70.48%)
		Village	62 (29.52%)
Civil status		Alone	47 (22.38%)
		Being in the relationship	163 (77.62%)
Education		Basic	15 (7.14%)
		Vocational	61 (29.05%)
		Secondary	81 (38.57%)
		Higher	53 (25.24%)
Professional activity		Employed	38 (18.10%)
		Retiree	102 (48.57%)
		Pensioner	66 (31.43%)
		Unemployed	4 (1.90%)
Comorbidities	Diabetes	No	161 (76.67%)
		Yes	49 (23.33%)
	Hypercholesterolemia	No	188 (89.52%)
		Yes	22 (10.48%)
	Hypertension	No	39 (18.57%)
		Yes	171 (81.43%)
	Rheumatic diseases	No	178 (84.76%)
		Yes	32 (15.24%)
	Other	No	170 (80.95%)
		Yes	40 (19.05%)
Blood concentration of tacrolimus < 5 mg or ciclosporin < 100 mg		Never	79 (37.62%)
		Rare	50 (23.81%)
		Sometimes	33 (15.71%)
		Often	24 (11.43%)
		Very often	24 (11.43%)

Abbreviations: SD, standard deviation; *n*, number of participants.

**Table 3:** ARMS overall adherence: simple linear regression and multiple regression analyses.

Feature			Simple Linear Model				Multiple Model			
			P	95% CI		*p*	P	95% CI		*p*
Gender		Women	ref.				ref.			
		Men	0.092	−0.415	0.6	0.722	0.133	−0.431	0.698	0.644
Age		(years)	0.012	−0.007	0.031	0.219	0.017	−0.014	0.049	0.285
Place of residence		City	ref.				ref.			
		Village	−0.709	−1.256	−0.162	0.012 *	−0.733	−1.388	−0.079	0.029 *
Civil status		Alone	ref.				ref.			
		Being in a relationship	−0.018	−0.625	0.59	0.954	−0.086	−0.785	0.612	0.809
Education		Primary	ref.				ref.			
		Vocational	−0.439	−1.494	0.616	0.415	−0.779	−1.918	0.36	0.182
		Secondary	0.015	−1.014	1.044	0.978	−0.443	−1.565	0.679	0.44
		Higher	0.125	−0.946	1.195	0.82	−0.503	−1.745	0.74	0.429
Professional activity		Employed	ref.				ref.			
		Retiree	−0.34	−1.036	0.356	0.339	−0.662	−1.649	0.325	0.19
		Pensioner	−0.39	−1.136	0.356	0.307	−0.446	−1.307	0.416	0.312
		Unemployed	−1.526	−3.451	0.399	0.122	−0.89	−2.937	1.156	0.395
Time since transplant		(years)	0.022	−0.013	0.057	0.213	0.024	−0.018	0.066	0.262
Comorbidities	Diabetes	No	ref.				ref.			
		Yes	−0.34	−0.937	0.257	0.266	−0.407	−1.054	0.24	0.219
	Hypercholesterolemia	No	ref.				ref.			
		Yes	0.375	−0.45	1.201	0.374	−0.163	−1.089	0.762	0.73
	Hypertension	No	ref.				ref.			
		Yes	0.084	−0.567	0.735	0.81	0.003	−0.742	0.748	0.993
	Rheumatic diseases	No	ref.				ref.			
		Yes	−0.137	−0.841	0.568	0.704	−0.363	−1.178	0.452	0.384
	Other	No	ref.				ref.			
		Yes	−0.104	−0.749	0.54	0.751	0.1	−0.6	0.81	0.78
Blood concentration of tacrolimus < 5 mg or ciclosporin < 100 mg		Never	ref.				ref.			
		Rare	0.619	−0.042	1.28	0.068	0.388	−0.348	1.124	0.303
		Sometimes	0.095	−0.664	0.853	0.807	−0.064	−0.884	0.756	0.879
		Often	0.519	−0.334	1.372	0.234	0.553	−0.389	1.495	0.252
		Very often	0.061	−0.792	0.913	0.889	−0.304	−1.276	0.668	0.541
AIS		[points]	−0.035	−0.066	−0.005	0.025 *	−0.016	−0.06	0.027	0.464
HADS	Anxiety	[points]	0.035	−0.033	0.102	0.315	−0.047	−0.149	0.055	0.371
	Depression	[points]	0.064	−0.012	0.141	0.101	0.06	−0.075	0.195	0.387
WHOQoL-BREF	Perception of quality of life	[points]	−0.19	−0.514	0.134	0.252	−0.059	−0.46	0.343	0.775
	Perception of health	[points]	−0.251	−0.495	−0.006	0.046 *	−0.098	−0.413	0.217	0.543
	Physical dimension	[points]	−0.108	−0.201	−0.014	0.025 *	−0.014	−0.181	0.154	0.873
	Psychological dimension	[points]	−0.113	−0.22	−0.006	0.039 *	0.032	−0.169	0.233	0.754
	Social dimension	[points]	−0.052	−0.146	0.042	0.282	0.106	−0.037	0.25	0.148
	Environmental dimension	[points]	−0.167	−0.285	−0.048	0.006 *	−0.167	−0.35	0.016	0.075
TFI	Total score	[points]	0.094	0.004	0.185	0.041 *	0.07	−0.051	0.192	0.257
	Physical components	[points]	0.076	−0.04	0.191	0.2				
	Psychological components	[points]	0.228	−0.027	0.483	0.081				
	Social components	[points]	0.315	−0.055	0.685	0.097				
MMSE		[points]	−0.008	−0.098	0.083	0.865	0.049	−0.059	0.157	0.371

Abbreviations: P, parameter; 95% CI, confidence interval; *p*, statistical significance, ARMS; Adherence to Refills and Medications Scale; AIS, Acceptance of Illness Scale; HADS, Hospital Anxiety and Depression Scale; WHOQoL-BREF, World Health Organization Quality of Life—Short Form; TFI, Tilburg Frailty Indicator; and MMSE, Mini-Mental State Examination. Notes: * Statistical significance (*p* < 0.05).

**Table 4:** ARMS taking medications subscale: simple linear regression and multiple regression analysis.

Feature			Simple Linear Model				Multiple Model			
			P	95% CI		*p*	P	95% CI		*p*
Gender		Women	ref.				ref.			
		Men	0.169	−0.193	0.531	0.361	0.204	−0.194	0.602	0.316
Age		(years)	0.004	−0.009	0.018	0.526	0.007	−0.015	0.029	0.541
Place of residence		City	ref.				ref.			
		Village	−0.37	−0.763	0.024	0.067	−0.285	−0.746	0.176	0.228
Civil status		Alone	ref.				ref.			
		Being in a relationship	0.122	−0.312	0.555	0.583	0.147	−0.345	0.64	0.558
Education		Primary	ref.				ref.			
		Vocational	0.259	−0.492	1.01	0.5	0.08	−0.723	0.883	0.845
		Secondary	0.59	−0.142	1.323	0.116	0.323	−0.467	1.114	0.424
		Higher	0.574	−0.189	1.336	0.142	0.15	−0.725	1.026	0.737
Professional activity		Employed	ref.				ref.			
		Retiree	−0.185	−0.683	0.314	0.468	−0.173	−0.869	0.523	0.627
		Pensioner	−0.036	−0.57	0.498	0.895	−0.029	−0.636	0.578	0.925
		Unemployed	−0.763	−2.142	0.615	0.279	−0.382	−1.824	1.06	0.604
Time since transplant		(years)	0.022	−0.003	0.046	0.089	0.028	−0.002	0.057	0.065
Comorbidities	Diabetes	ref.				ref.				
		−0.286	−0.712	0.14	0.19	−0.4	−0.856	0.056	0.087	0.219
	Hypercholesterolemia	ref.				ref.				
		0.81	0.219	1.38	0.007 *	0.651	−0.001	1.303	0.052	0.73
	Hypertension	ref.				ref.				
		0.291	−0.172	0.755	0.219	0.22	−0.305	0.745	0.413	0.993
	Rheumatic diseases	ref.				ref.				
		0.047	−0.456	0.55	0.855	−0.205	−0.779	0.369	0.485	0.384
	Other	ref.				ref.				
		0.157	−0.303	0.617	0.503	0.27	−0.224	0.763	0.285	0.78
Blood concentration of tacrolimus < 5 mg or ciclosporin < 100 mg		Never	ref.				ref.			
		Rare	0.221	−0.255	0.697	0.363	0.082	−0.437	0.6	0.758
		Sometimes	0.178	−0.367	0.723	0.523	0.136	−0.442	0.714	0.644
		Often	0.273	−0.341	0.886	0.385	0.244	−0.419	0.908	0.471
		Very often	0.148	−0.466	0.761	0.638	−0.006	−0.691	0.679	0.986
AIS		[points]	−0.024	−0.046	−0.002	0.037 *	−0.012	−0.042	0.019	0.458
HADS	Anxiety	0.026	−0.022	0.074	0.298	−0.048	−0.119	0.024	0.196	0.371
	Depression	0.051	−0.004	0.105	0.07	0.061	−0.034	0.156	0.211	0.387
WHOQoL-BREF	Perception of quality of life	−0.096	−0.328	0.136	0.417	−0.034	−0.317	0.249	0.816	0.775
	Perception of health	−0.122	−0.298	0.053	0.174	0.073	−0.15	0.295	0.523	0.543
	Physical dimension	−0.09	−0.157	−0.024	0.008 *	−0.09	−0.208	0.028	0.138	0.873
	Psychological dimension	−0.046	−0.122	0.031	0.246	0.053	−0.089	0.195	0.464	0.754
	Social dimension	−0.022	−0.089	0.045	0.517	0.051	−0.05	0.152	0.326	0.148
	Environmental dimension	−0.073	−0.158	0.013	0.096	−0.038	−0.167	0.091	0.56	0.075
TFI	Total score	0.05	−0.015	0.115	0.132	0.054	−0.032	0.139	0.22	0.257
	Physical components	0.038	−0.045	0.12	0.371					
	Psychological components	0.193	0.011	0.374	0.039 *					
	Social components	0.039	−0.227	0.305	0.774					
MMSE		[points]	0.047	−0.017	0.111	0.153	0.084	0.008	0.16	0.032 *

Abbreviations: P, parameter; 95% CI, confidence interval; *p*, statistical significance, ARMS; Adherence to Refills and Medications Scale; AIS, Acceptance of Illness Scale; HADS, Hospital Anxiety and Depression Scale; WHOQoL-BREF, World Health Organization Quality of Life—Short Form; TFI, Tilburg Frailty Indicator; and MMSE, Mini-Mental State Examination. Notes: * Statistical significance (*p* < 0.05).

**Table 5:** ARMS taking medications and refilling prescriptions: simple linear regression and multiple regression analyses.

Feature			Simple Linear Model				Multiple Model			
			P	95% CI		*p*	P	95% CI		*p*
Gender		Women	ref.				ref.			
		Men	−0.077	−0.431	0.278	0.673	−0.071	−0.463	0.322	0.725
Age		(years)	0.008	−0.006	0.021	0.267	0.01	−0.012	0.032	0.358
Place of residence		City	ref.				ref.			
		Village	−0.34	−0.725	0.046	0.086	−0.448	−0.904	0.007	0.055
Civil status		Alone	ref.				ref.			
		Being in a relationship	−0.14	−0.564	0.285	0.52	−0.234	−0.719	0.252	0.347
Education		Primary	ref.				ref.			
		Vocational	−0.698	−1.434	0.038	0.064	−0.859	−1.651	−0.067	0.035 *
		Secondary	−0.575	−1.293	0.143	0.118	−0.766	−1.547	0.014	0.056
		Higher	−0.449	−1.196	0.298	0.24	−0.653	−1.517	0.211	0.14
Professional activity		Employed	ref.				ref.			
		Retiree	−0.155	−0.642	0.331	0.532	−0.489	−1.176	0.197	0.164
		Pensioner	−0.354	−0.876	0.167	0.185	−0.417	−1.016	0.183	0.175
		Unemployed	−0.763	−2.109	0.583	0.268	−0.508	−1.931	0.915	0.485
Time since transplant		(years)	0.001	−0.024	0.025	0.964	−0.004	−0.033	0.025	0.794
Comorbidities	Diabetes	ref.				ref.				
		−0.054	−0.473	0.364	0.81	−0.007	−0.457	0.443	0.976	0.219
	Hypercholesterolemia	ref.				ref.				
		−0.425	−1	0.15	0.149	−0.814	−1.458	−0.171	0.014 *	0.73
	Hypertension	ref.				ref.				
		−0.207	−0.662	0.247	0.372	−0.217	−0.735	0.301	0.413	0.993
	Rheumatic diseases	ref.				ref.				
		−0.184	−0.675	0.308	0.465	−0.158	−0.725	0.409	0.585	0.384
	Other	ref.				ref.				
		−0.262	−0.711	0.188	0.255	−0.17	−0.656	0.317	0.495	0.78
Blood concentration of tacrolimus < 5 mg or ciclosporin < 100 mg		Never	ref.				ref.			
		Rare	0.398	−0.064	0.86	0.093	0.306	−0.205	0.818	0.242
		Sometimes	−0.083	−0.613	0.446	0.758	−0.2	−0.77	0.37	0.493
		Often	0.246	−0.349	0.842	0.419	0.308	−0.347	0.964	0.357
		Very often	−0.087	−0.683	0.509	0.775	−0.298	−0.973	0.378	0.389
AIS		[points]	−0.012	−0.034	0.01	0.284	−0.005	−0.035	0.026	0.763
HADS	Anxiety	0.009	−0.038	0.056	0.709	0.001	−0.07	0.072	0.98	0.371
	Depression	0.014	−0.04	0.068	0.619	−0.001	−0.095	0.093	0.982	0.387
WHOQoL-BREF	Perception of quality of life	−0.094	−0.321	0.133	0.418	−0.025	−0.304	0.254	0.861	0.775
	Perception of health	−0.128	−0.3	0.044	0.145	−0.171	−0.39	0.049	0.129	0.543
	Physical dimension	−0.017	−0.084	0.049	0.605	0.076	−0.041	0.193	0.203	0.873
	Psychological dimension	−0.068	−0.142	0.007	0.078	−0.021	−0.161	0.119	0.77	0.754
	Social dimension	−0.029	−0.095	0.036	0.381	0.056	−0.044	0.155	0.276	0.148
	Environmental dimension	−0.094	−0.177	−0.011	0.028 *	−0.129	−0.256	−0.002	0.049 *	0.075
TFI	Total score	0.044	−0.019	0.108	0.17	0.017	−0.068	0.101	0.698	0.257
	Physical components	0.038	−0.043	0.119	0.357					
	Psychological components	0.035	−0.144	0.215	0.699					
	Social components	0.276	0.019	0.534	0.037 *					
MMSE		[points]	−0.055	−0.118	0.008	0.088	−0.034	−0.109	0.041	0.369

Abbreviations: P, parameter; 95% CI, confidence interval; *p*, statistical significance, ARMS; Adherence to Refills and Medications Scale; AIS, Acceptance of Illness Scale; HADS, Hospital Anxiety and Depression Scale; WHOQoL-BREF, World Health Organization Quality of Life—Short Form; TFI, Tilburg Frailty Indicator; and MMSE, Mini-Mental State Examination. Notes: * Statistical significance (*p* < 0.05).

## Data Availability

The data presented in this study are available on request from the corresponding author.
